# Trends in Gender Representation and Post-residency In-State Retention at Both an Academic Medical Center and in the United States

**DOI:** 10.7759/cureus.12269

**Published:** 2020-12-25

**Authors:** Kevin Chiang, Kimberly Lu, Molly Hartley, Zhexi Lu, Trishae Winters, David R Hallan, Surav M Sakya

**Affiliations:** 1 Pediatrics, Penn State College of Medicine, Hershey, USA; 2 Medicine, Penn State College of Medicine, Hershey, USA; 3 Neurosurgery, Penn State Health Milton S. Hershey Medical Center, Hershey, USA

**Keywords:** gender, retention rate, representation, pennsylvania, penn state, united states, residency, medical school

## Abstract

This study examines gender representation and in-state retention rates of practicing residency graduates from Pennsylvania State University (PSU), as well as at the national level. PSU and national data were collected from a PSU handbook and the Association of American Medical Colleges (AAMC), respectively. There were significant differences between male and female representation both at PSU and at the national level. Furthermore, there was a significant difference between male and female retention rates nationally. This study demonstrates a true gender discrepancy for graduates from PSU and at the national level. Moving forward, investigating potential causes of this discrepancy may help minimize gender differences.

## Introduction

In 1849, Elizabeth Blackwell became the first woman to graduate with a medical degree in the United States [1]. Since then, the proportion of female physicians comprising the physician workforce has been increasing. Currently, 35.2% of practicing physicians in the United States are female. In 2019, women comprised the majority of medical students for the first time, and in 2020, women made up 53.6% of matriculants to medical schools in the United States [2-3]. Although more women are entering the physician workforce, some specialties have noticeably skewed gender representations [4]. At a national level, obstetrics and gynecology (OBGYN) has the highest percentage of female representation, while orthopedic surgery has the highest percentage of men [5].

Several factors may be contributing to these gender discrepancies. For example, previous studies have suggested that women may not choose orthopedic surgery because of work/life balance, perceptions about strength, lack of mentorship, and discrimination [6-9]. In OBGYN, male physicians reported that their gender negatively affected patient volume [10]. Considering this vast problem, residency programs are attempting to minimize the gender divide because a more balanced gender representation diversifies perspectives and prepares more culturally competent physicians [11].

Gender also plays a role in the selection of post-residency practice locations. A prior study found that women are more likely than men to be influenced by issues related to spouses, flexible scheduling, childcare availability, and family leave [12]. These gender-related factors could impact in-state retention rates across different specialties.

The purpose of this study is to compare the gender representation and in-state retention rate of practicing residency graduates from both Pennsylvania State University (PSU) and at the national level. We hypothesize that there will be an uneven distribution of male and female residency graduates regarding both representation and in-state retention for PSU and at the national level.

## Materials and methods

This retrospective study examined data regarding PSU residency alumni and national data. PSU data were obtained from Penn State match lists, and national data were compiled from the Association of American Medical Colleges (AAMC) [13]. PSU match list's data were taken from all years for which there were data publicly available on the PSU website for each specialty. The AAMC data were produced in 2017 using information from the American Medical Association's Physician Masterfile from 2017. Tables and graphs were generated to compare PSU and national data. Data analysis was performed using paired t-tests with statistical significance set at p < 0.05. Tables and graphs were created to show gender distribution and in-state retention rates across different specialties both at PSU and nationally. Institutional review board (IRB) exemption was granted.

## Results

Gender of practicing physicians by specialty

**Figure 1:**
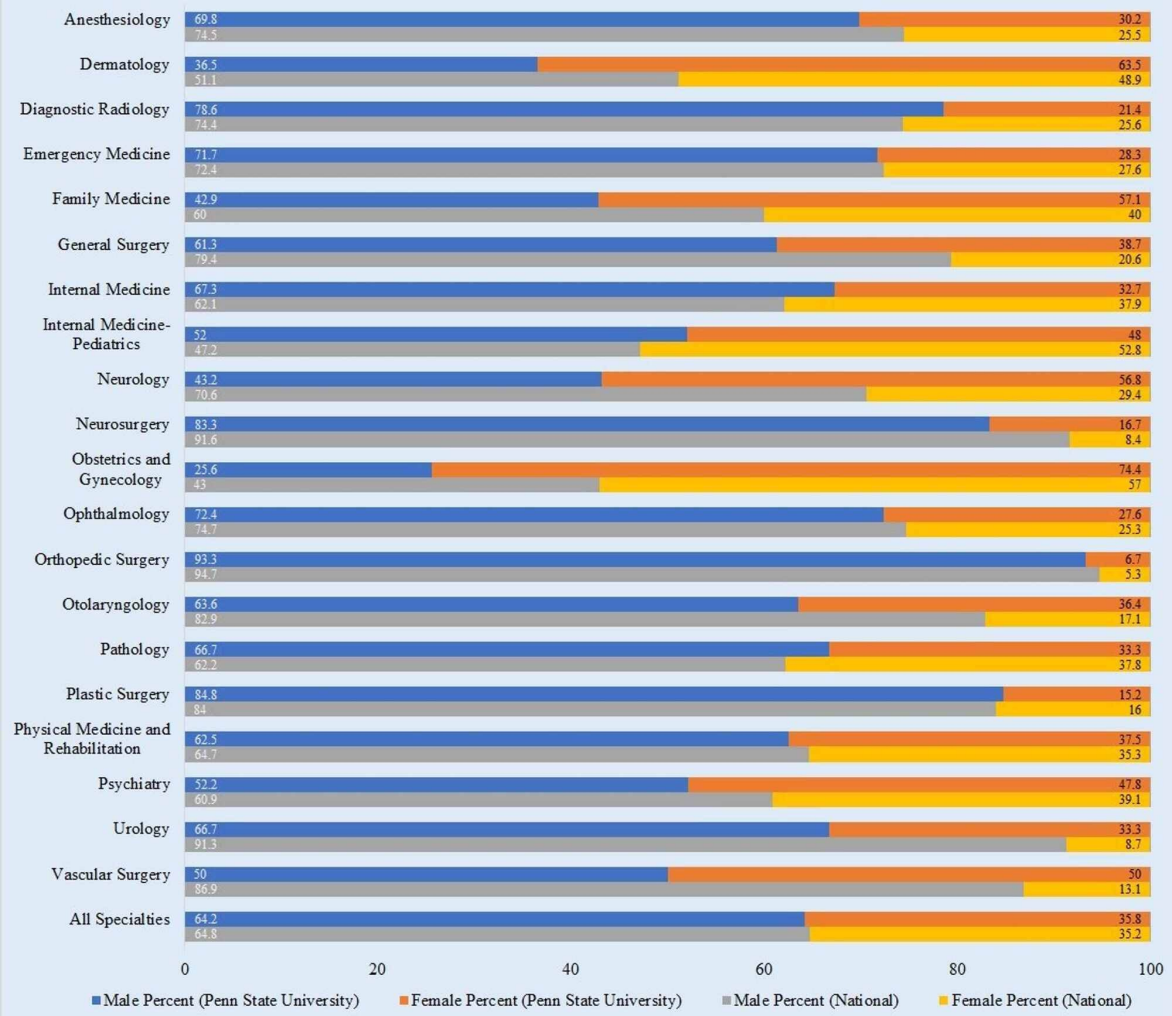
Gender of Practicing Physicians by Specialty – Comparison of PSU and National Data. PSU: Pennsylvania State University

Females comprise 35.8% of practicing PSU residency graduates and 35.2% of practicing physicians nationally (Table 1, Figure 1). PSU female physicians were most represented in OBGYN (74.4%), dermatology (63.5%), family medicine (57.1%), neurology (56.8%), and vascular surgery (50%). Nationally, female representation was highest in OBGYN (57%), internal medicine/pediatrics (Med/Peds) (52.8%), dermatology (48.9%), family medicine (40%), and psychiatry (39.1%). Females were, therefore, most represented in OBGYN both at PSU and nationally. Meanwhile, orthopedic surgery was the most male-dominated specialty, with 93.3% at PSU and 94.7% nationally. At PSU, male representation was higher in 15 out of the 20 specialties examined. Of these, the top five specialties with the greatest male representation were orthopedic surgery (93.3%), plastic surgery (84.8%), neurosurgery (83.3%), diagnostic radiology (78.6%), and ophthalmology (72.4%). In comparison, nationally, male representation was highest in orthopedic surgery (94.7%), neurosurgery (91.6%), urology (91.3%), vascular surgery (86.9%), and plastic surgery (84%). A paired t-test revealed statistical significance between male and female representation by specialty for both PSU (p = 0.037) and the national data (p = 0.000138).

**Table 1:** Gender of Practicing Physicians by Specialty – Comparison of PSU and National Data. PSU: Pennsylvania State University

#	Residency Programs	Male Percent (PSU)	Female Percent (PSU)	Male Percent (National)	Female Percent (National)
1	Anesthesiology	69.8	30.2	74.5	25.5
2	Dermatology	36.5	63.5	51.1	48.9
3	Diagnostic Radiology	78.6	21.4	74.4	25.6
4	Emergency Medicine	71.7	28.3	72.4	27.6
5	Family Medicine	42.9	57.1	60.0	40.0
6	General Surgery	61.3	38.7	79.4	20.6
7	Internal Medicine	67.3	32.7	62.1	37.9
8	Internal Medicine-Pediatrics	52.0	48.0	47.2	52.8
9	Neurology	43.2	56.8	70.6	29.4
10	Neurosurgery	83.3	16.7	91.6	8.4
11	Obstetrics and Gynecology	25.6	74.4	43.0	57.0
12	Ophthalmology	72.4	27.6	74.7	25.3
13	Orthopedic Surgery	93.3	6.7	94.7	5.3
14	Otolaryngology	63.6	36.4	82.9	17.1
15	Pathology	66.7	33.3	62.2	37.8
16	Plastic Surgery	84.8	15.2	84.0	16.0
17	Physical Medicine and Rehabilitation	62.5	37.5	64.7	35.3
18	Psychiatry	52.2	47.8	60.9	39.1
19	Urology	66.7	33.3	91.3	8.7
20	Vascular Surgery	50.0	50.0	86.9	13.1
ALL	All Specialties	64.2	35.8	64.8	35.2

Physician retention in state of residency training by gender and specialty

The top five specialties for in-state retention for male physicians nationally were family medicine (63.5%), psychiatry (61.7%), vascular surgery (60.2%), pathology (58.4%), and internal medicine (57.6%) (Table 2, Figure 2). The top five specialties for female physicians nationally were psychiatry (67.1%), family medicine (65.5%), pathology (61.3%), internal medicine-pediatrics (61.3%), and internal medicine (59.4%). At PSU, the top five specialties for male physicians were internal medicine (70%), vascular surgery (50%), psychiatry (41.7%), family medicine (41.7%), and dermatology (37%). For female PSU physicians, the top five specialties were psychiatry (54.5%), vascular surgery (50%), ophthalmology (50%), internal medicine (50%), and family medicine (50%). Overall, female physicians were more likely to stay in the state where they trained, both at the national level (58.3% for females and 51.4% for males) and at PSU (39% for females and 30.3% for males). A paired t-test showed no significant difference between male and female retention rates at PSU (p = 0.174), but it revealed a significant difference nationally (p = 0.000567).

**Table 2:** Physician Retention in State of Residency Training by Gender and Specialty – Comparison of PSU and National Data. PSU: Pennsylvania State University

#	Residency Programs	National				PSU			
		Male		Female		Male		Female	
		In State	Out of State	In State	Out of State	In State	Out of State	In State	Out of State
1	Anesthesiology	53.6	46.4	56.6	43.4	36.7	63.3	46.7	53.3
2	Dermatology	48.1	51.9	53.6	46.4	37.0	63.0	46.8	53.2
3	Diagnostic Radiology	47.9	52.1	52.4	47.6	9.1	90.9	33.3	66.7
4	Emergency Medicine	51.3	48.7	54.8	45.2	24.2	75.8	30.8	69.2
5	Family Medicine	63.5	36.5	65.5	34.5	41.7	58.3	50.0	50.0
6	General Surgery	50.7	49.3	51.8	48.2	23.7	76.3	29.2	70.8
7	Internal Medicine	57.6	42.4	59.4	40.6	70.0	30.0	50.0	50.0
8	Internal Medicine-Pediatrics	57.1	42.9	61.3	38.7	28.2	71.8	36.1	63.9
9	Neurology	50.8	49.2	52.9	47.1	26.3	73.7	36.0	64.0
10	Neurosurgery	39.8	60.2	49.0	51.0	20.0	80.0	0	100
11	Obstetrics and Gynecology	47.6	52.4	53.2	46.8	30.0	70.0	41.4	58.6
12	Ophthalmology	36.9	63.1	44.9	55.1	33.3	66.7	50.0	50.0
13	Orthopedic Surgery	41.8	58.2	42.1	57.9	21.4	78.6	16.7	83.3
14	Otolaryngology	40.3	59.7	46.0	54.0	21.4	78.6	0	100
15	Pathology	58.4	41.6	61.3	38.7	25.0	75.0	12.5	87.5
16	Plastic Surgery	51.4	48.6	57.6	42.4	21.4	78.6	30.0	70.0
17	Physical Medicine and Rehabilitation	44.0	56.0	51.2	48.8	20.0	80.0	33.3	66.7
18	Psychiatry	61.7	38.3	67.1	32.9	41.7	58.3	54.5	45.5
19	Urology	42.5	57.5	39.2	60.8	0	100	0	100
20	Vascular Surgery	60.2	39.8	56.9	43.1	50.0	50.0	50.0	50.0
ALL	All Specialties	51.4	48.6	58.3	41.7	30.3	69.7	39.0	61.0

**Figure 2:**
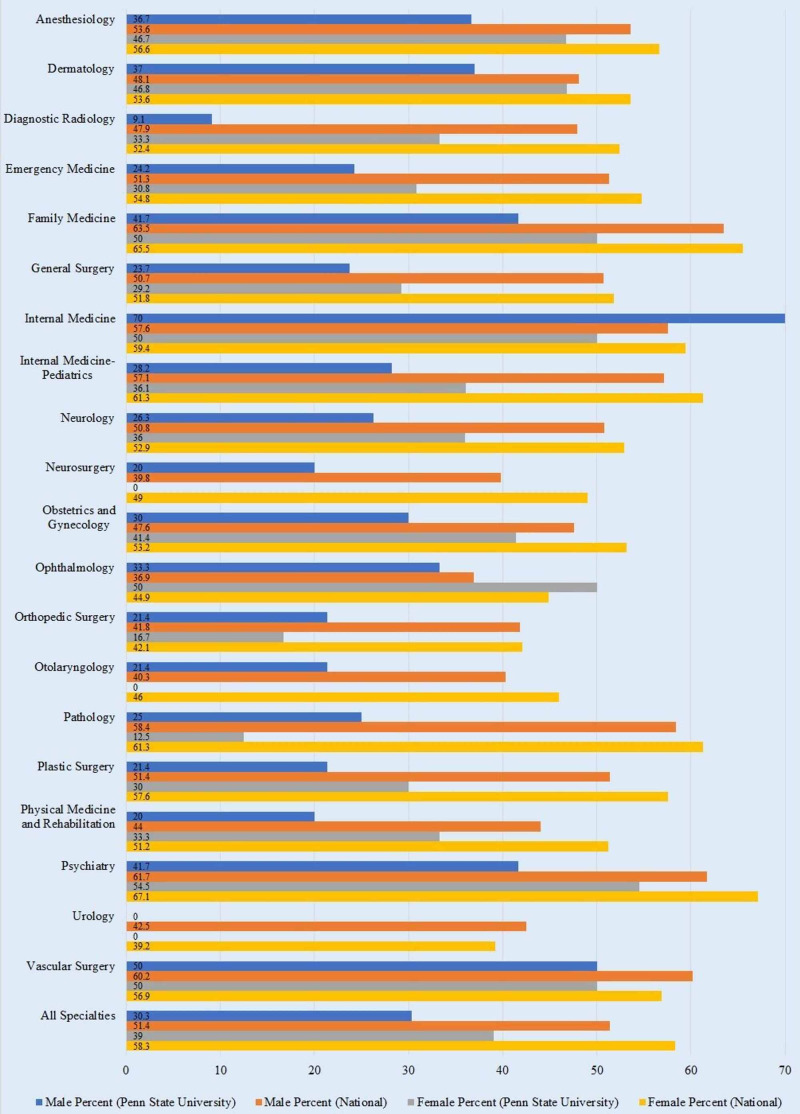
Physician Retention in State of Residency Training by Gender and Specialty – Comparison of PSU and National Data. PSU: Pennsylvania State University

## Discussion

The results support our hypothesis of an uneven gender distribution for residency graduates at PSU and the national level. One possible explanation for this is work/life balance. According to the results of this study, the relative representation of women was generally higher in medicine-based specialties that are typically associated with better schedules and work/life balance, while the relative representation of men was generally higher in surgical specialties that typically require longer work hours. However, at PSU, the relative representation of women was highest in OBGYN and vascular surgery. This likely illustrates that work/life balance is certainly not the only factor influencing gender discrepancies in residency programs. Additionally, lack of mentorship may deter potential applicants, as studies have shown for females considering surgical careers [6-8]. Future studies are needed to further investigate factors that may contribute to these gender distribution discrepancies. The ratio of female to male residents was also higher at PSU for certain specialties, such as dermatology, compared to the ratio seen at the national level. Differences in sample size may help explain the differences in these findings. However, it is unknown whether there are aspects specific to the structure, culture, or benefits of certain residency programs that could also explain these differences. Future qualitative investigations may provide a clearer picture of what factors contribute to these findings. Identification of such factors could be used to design and implement changes that may help reduce gender discrepancies in representation.

There were significant differences between male and female retention rates nationally. One potential explanation is that women are more likely to be influenced by factors such as scheduling flexibility and family leave when considering practice locations [12]. Furthermore, based on this study, males are more likely to choose surgical specialties, which may influence decisions to move out of state due to fellowship and job opportunities. Interestingly, no significant difference was found between genders for retention rate at PSU. This may be due to location-specific factors related to Hershey or Pennsylvania that drive individuals, both male and female, to remain in the state. To our knowledge, this has not specifically been studied in the literature. Future surveys of PSU residency graduates could help to identify some of these factors. Alternatively, this finding may be due to a relatively smaller sample size at PSU in comparison to that used to produce the national data. Future studies should investigate specific factors that may contribute to the discrepancy in gender distribution and the correlation between surgical specialties and in-state retention.

Limitations

Pediatrics was excluded because PSU’s pediatric residency data were not fully available. Making such data publicly available or obtaining pediatric-specific data would allow a more complete characterization of differences in gender distribution. Additionally, current information on some resident graduates was not publicly available, and those individuals were excluded from the data analyzed. Furthermore, the number of years for which there were available PSU match list data varied depending on the specialty. Given that this is a single-institution study, the results from PSU are not necessarily generalizable to other programs or regions around the country.

## Conclusions

This study suggests that gender discrepancy continues to exist both at PSU and the national level. The relative representation of women was higher in medical specialties, and the relative representation of men was higher in surgical specialties. There was a notable difference between genders in retention rates nationally. Further investigation of potential factors that may explain why this gender breakdown exists can help minimize gender disparities in the future at both an institutional and national level, as well as justify changes that may encourage medical students to pursue specialties they might otherwise not consider. Future studies may also examine differences in gender breakdown for individual specialties that have not been previously studied extensively in the literature.
